# Association of Children’s Dietary Inflammatory Index with Depression and Anxiety Symptoms in Adolescents: Mediating Role of Inflammation and Cardiometabolic Risk Factors

**DOI:** 10.31083/AP38791

**Published:** 2025-02-28

**Authors:** Kezban Şahin, Hülya Yardımcı, Murat Açık, Alkım Öden Akman, Fadime Yüksel

**Affiliations:** ^1^Department of Nutrition and Dietetics, Faculty of Health Sciences, Bandırma Onyedi Eylül University, 10200 Balıkesir, Turkey; ^2^Department of Nutrition and Dietetics, Faculty of Health Sciences, Ankara University, 06290 Ankara, Turkey; ^3^Department of Nutrition and Dietetics, Faculty of Health Sciences, Fırat University, 23200 Elazığ, Turkey; ^4^Department of Pediatric & Adolescent Medicine, Children Hospital, Ankara City Hospital, 06700 Ankara, Turkey; ^5^Department of Social Pediatrics, Children Hospital, Ankara City Hospital, 06700 Ankara, Turkey

**Keywords:** C-DII, depression, anxiety, inflammation, adolescents

## Abstract

**Background:**

Evidence is scarce on the mechanisms involved in the relationship between dietary inflammatory index and mental health in adolescents. This study aimed to assess the association between children-DII (C-DII) and depressive and anxiety disorder symptoms in adolescents and to explore whether inflammation and cardiometabolic risk factors mediate this association.

**Methods:**

The study was conducted at the Ankara City Hospital Pediatrics Polyclinic and 304 adolescents. In cross-sectional study, adolescents were asked general information questions. Anthropometric measurements were performed and some biochemical parameters and inflammation (C-reactive protein (CRP)) were obtained. The C-DII score was calculated from 24-h dietary recalls. Depression and anxiety levels of the participants were assessed by self-report. Structural equation modelling analyzed how cardiometabolic risk factors and inflammation mediate the relationship between mental health and dietary inflammation.

**Results:**

C-DII scores were positively associated with depression and anxiety score (β [95% confidence interval (CI)] = 0.224 [0.08–0.25] for depression; 0.923 [0.04–1.67] for anxiety). Except for dietary inflammation with anxiety in girls, these relationships remained statistically significant in all subgroups by sex. It was determined that CRP partially mediated the relationship between dietary inflammation and depression and anxiety. It was determined that body mass index (BMI)-z score and waist circumference (WC) mediated the relationship between dietary inflammation and depression scores.

**Conclusions:**

Our findings indicate that the higher pro-inflammatory potential of diet is associated with a higher risk of depression and anxiety, and this association may be mediated by CRP for depression and anxiety, WC, and BMI-z score for only depression. Further research is required to verify our findings and clarify the latent mechanism in larger populations.

## Main Points

1. In adolescent participants, a higher pro-inflammatory diet was associated with increased depression and anxiety.

2. The association between pro-inflammatory diet and anxiety was found to be stronger compared to depression.

3. The relationship between Children’s dietary inflammatory index and anxiety was mediated by C-reactive protein and waist circumference.

4. However, the effect was found to be very poor in the results from the mediation analysis. Therefore, longitudinal studies, potentially minimizing the effects of confounders, are needed in the future.

## 1. Introduction

Mental disorders (depression and anxiety etc.) are an important public health problem that is thought to affect more than 300 million people worldwide and have a serious burden on socioeconomic costs [1]. In many countries, mental problems have the highest prevalence in young adults and adolescents, and one in five children or teenagers worldwide report having one of these problems [2]. Internalization problems, which are defined as symptoms of emotional problems such as depression and anxiety, are frequently reported among those under the age of 18, and the proportion of young people adopting these symptoms has increased over time [3]. Early diagnosis and treatment of mental disorders in children and adolescents play a critical role in preventing the continuing morbidity and mortality associated with these conditions throughout life.

Although the major biological mechanisms in the etiology of depression and anxiety are hypothalamic–pituitary–adrenal (HPA) imbalances and the serotonin hypothesis in adolescents, it has recently been suggested that inflammation may play an important role in depression as well as in asthma, cardiovascular diseases, obesity, and inflammatory bowel diseases [4, 5]. In systematic reviews and meta-analyses conducted on youth and adolescents, the levels of peripheral cytokines such as interleukin (IL)-6, C-reactive protein (CRP), and tumor necrosis factor alpha (TNF-α) were found to be high in patients with a clinical diagnosis of depression or anxiety, probably because of the effect of psychological and physiological stress on the immune system [5, 6]. In addition, the inflammatory state can negatively affect the responses of escitalopram and nortriptyline, which are important pharmacological agents in mental problems such as depression and anxiety, thus causing the disease to settle permanently [7]. Overall, these studies actually highlight the potential to reduce inflammation through anti-inflammatory interventions and thus reduce the risk of mental problems.

The role of nutrition in mental health, known as nutritional psychiatry, has become the focus of attention in recent years. Childhood and adolescence are the stages of rapid growth and development of the brain, and the effectiveness of nutrients such as omega-3 fatty acids on cognition is well known [3]. Several possible mechanisms may explain depression through biological mechanisms such as dietary patterns, inflammation, gut-brain axis, and brain-derived neurotrophic factor (BDNF) [5]. Longitudinal and cross-sectional observational studies have shown that a high-quality dietary intake or adherence to healthy dietary patterns, such as the Mediterranean diet, is associated with a lower likelihood of depression and anxiety in adolescents [8] and adults [9]. In addition, evidence from randomized controlled trials suggests that dietary interventions aimed at reducing fat intake and promoting weight loss reduce depressive symptoms but not anxiety symptoms [10]. However, a population-based dietary inflammatory index (DII) derived from the literature was developed to assess the inflammatory potential of an individual’s diet [11]. Data obtained from studies on different target groups, such as university students, middle-aged women, nurses, and the general adult population, supported the relationship between increased DII and depression [12, 13].

Although the relationship between DII and depression has been investigated in adults, research examining this relationship in adolescents is limited. Akbaraly* et al*. [14] reported that high DII scores (indicating a pro-inflammatory diet) were positively associated with greater circulating levels of IL-6 and CRP, as well as a higher risk of recurrent depressive symptoms in adult women of the Whitehall II study. Jorgensen* et al*. [15] observed that adult depressed individuals in the NHANES 2007–2012 cohort had higher CRP concentrations, which were positively associated with dietary inflammation. In addition, there is persuasive evidence that high hs-CRP concentrations are associated with increased cardiometabolic risk [16]. In adolescents, unhealthy diet patterns cause obesity and an increase in metabolic risk factors, thus increasing the symptoms of depression and anxiety. Unhealthy diet patterns (such as consuming the western diet etc.) have been shown to increase the levels of peripheral inflammatory markers such as IL-6 and CRP, as well as blood glucose and lipid profile levels, which are associated with the development of depression symptoms [17]. However, the association of DII with depressive symptoms and inflammation, cardiometabolic risk in adolescents has not been well elucidated. Research for this purpose is limited and shows inconsistent results [12, 18]. Furthermore, we believe that it is crucial to consider sex-specific mechanisms of progression and development of metabolic risk factors, potentially revealing sex-specific interventions, as girls and boys have genetic and biological differences.

Therefore, this study aims (1) to evaluate the relationship between DII index score and depression and anxiety and (2) to investigate whether possible inflammation markers (CRP) and cardiometabolic risk factors (anthropometric measurements and biochemical parameters) play a mediating role in this relationship in adolescents.

## 2. Materials and Methods

### 2.1 Study Population

This research was conducted with 304 adolescents aged 10–14 years, who have Turkish language proficiency, in the Ankara City Hospital Pediatrics and Diseases/Healthy Child Polyclinics and General Pediatrics Polyclinic between January and June 2021. We excluded participants who used cigarettes and alcohol, had any major or metabolic disease diagnosed by a physician, used antidepressants, had been diagnosed with eating disorders, and were unwilling adolescents. Before the study, general information about the aim of the study was provided to the adolescents and their parents, and we included those who signed the voluntary consent form. A data collection form was applied to the adolescents using the face-to-face interview technique. The data collection form included demographic information, some anthropometric measurements, Children’s Depression Inventory (CDI), Social Anxiety Scale for Children-Revised Form (SASC-R), and 24-h dietary recall interview form. A preliminary study was conducted with ten people to determine the deficiencies in the research. After the deficiencies were corrected, data collection began.

### 2.2 Anthropometric Measurements

Anthropometric measurements of adolescents were performed by the researchers during the interview in accordance with the method. Body weight was measured as much as possible when individuals were hungry in the morning and after defecation, in a fixed position, with hands and arms on both sides, in accordance with the measurement technique. Height (Ht) was assessed to the nearest 0.1 cm with each participant standing without shoes and the shoulders positioned against a stadiometer. Body mass index (BMI) was calculated using the standard formula (kg/m^2^). Waist circumference was measured to the nearest 0.1 cm using a flexible, nonstretchable tape and at the midpoint between the lowest rib and the iliac crest, with the students standing and breathing out. The World Health Organization (WHO) AnthroPlus program was used to calculate the BMI z-scores of adolescents by age. In its evaluation, “WHO 2007 reference values for children aged 5–19 years” were used [19]. The waist-to-height ratio was calculated as waist/height. The waist-to-height ratio was evaluated according to the following cutoffs: normal (<0.5) and abdominal obesity (≥0.5) [20].

### 2.3 Biochemical Assessments

Blood fasting glucose (BFG), low-density lipoprotein cholesterol (LDL-C), high-density lipoprotein cholesterol (HDL-C), C-reactive protein (CRP), triglyceride, and albumin values, which are some of the biochemical parameters of adolescents routinely checked in the outpatient clinic, were directly demanded from the parents themselves. Ankara City Hospital Central Laboratory reference values were used to evaluate the blood findings of the parents who allowed the use of biochemical parameters.

### 2.4 Psychological Assessment of Depression and Anxiety

The Children’s Depression Inventory (CDI) is a reliable and well-tested symptom-focused scale that measures depression symptoms in children and adolescents [21]. The items of this scale, which has a triple Likert structure and consists of 27 items, are scored as zero, one, and two, and depressive symptoms increase with the score. The Turkish validity and reliability study of the scale was performed by Öy [22].

The Social Anxiety Scale for Children-Revised (SASC-R) was developed to measure social anxiety in children and adolescents and is based on self-reporting. Consisting of ten questions, this scale was revised in 1993 and turned into a scale of 18 questions. This scale is in the form of a five-point Likert scale, and the score range is 18–90 [23]. As the score obtained from the scale increases, the level of social anxiety also increases. The validity and reliability study of the Turkish version of the scale was conducted by Demir* et al*., in 2000 [24]. The Cronbach Alpha internal consistency reliability coefficient of the scale was 0.81 [24].

### 2.5 Evaluation of 24-Hour Dietary Intake Record and Calculation of the Dietary Inflammatory Index and the Dietary Inflammatory Index for Children

To determine the food consumption status of the adolescents, a 24-h dietary recall interview form was completed. The data obtained from a daily food consumption record were evaluated and analyzed using nutrient analysis software (BeBiS), and the average daily energy intake and macro- and micronutrient intake were analyzed.

The Dietary Inflammatory Index (DII) is an index created to evaluate the inflammatory potential of the diet. The DII was calculated on energy, macronutrients, and micronutrients obtained from a 24-h dietary recall interview form of adolescents. A total of 45 foods and nutrients were used to calculate the Dietary Inflammatory Index. Calculation of the DII score of the adolescents participating in the research was carried out as follows: First, the z-score values from the intakes of the nutrients/nutrients of the adolescents (z-score value = [(Daily consumption amount of the mentioned nutrient parameter by adolescents-average global daily intake) / the standard value of the food parameter in question. deviation value]) were calculated, and the calculated z-score values were converted to percentile score. Then, percentile values determined for each nutritional parameter were multiplied with the customized full inflammatory effect score specified in the table; The total DII score of the adolescents was obtained by summing these values, which were calculated one by one for each nutrient or nutrient [11].

The calculation of the Dietary Inflammatory Index for Children (C-DII), which is appropriate for use in adolescents, was performed as previously described in the literature. The C-DII is a literature-based index, and the methods used in the development of DII were also used in its development [25]. Nutritional parameters used in the calculation: energy, carbohydrate, protein, total fat, alcohol, fiber, cholesterol, saturated fat, monounsaturated fatty acids (MUFA), polyunsaturated fatty acids (PUFA), vitamin A, vitamin E, vitamin C, vitamin D, β carotene, thiamine, riboflavin, niacin, vitamin B6, folic acid, vitamin B12, iron, magnesium, zinc, and selenium. C-DII has been developed to reflect the diversity of standardized dietary patterns around the world [25].

### 2.6 Other Variables

In this part of the questionnaire, the age, gender, health status of the adolescents, and socioeconomic status of the family were examined.

### 2.7 Statistical Analysis

Data analysis was performed using IBM Statistical Package Software for Social Science (SPSS) version 26 (SPSS Inc., Chicago, IL, USA) and IBM AMOS version 22 for Windows. Means, standard errors, and percentages were used to illustrate quantitative and qualitative variables to compare the difference between categorical variables, the chi-square test was used, and continuous variables were tested using Analysis of Variance (ANOVA). C-DII scores were categorized into textiles [Tertile 1 (Anti-inflammatory), Tertile 2, Tertile 3 (Pro-inflammatory)]. Moreover, linear regression analyses were performed to calculate the β-coefficient of the association between the dietary inflammation index and depression and anxiety status by gender after the necessary criteria were provided. The β coefficient, explanation coefficient (R^2^), and 95% confidence interval (CI) are presented in the linear regression analysis.

**Fig. 1.**
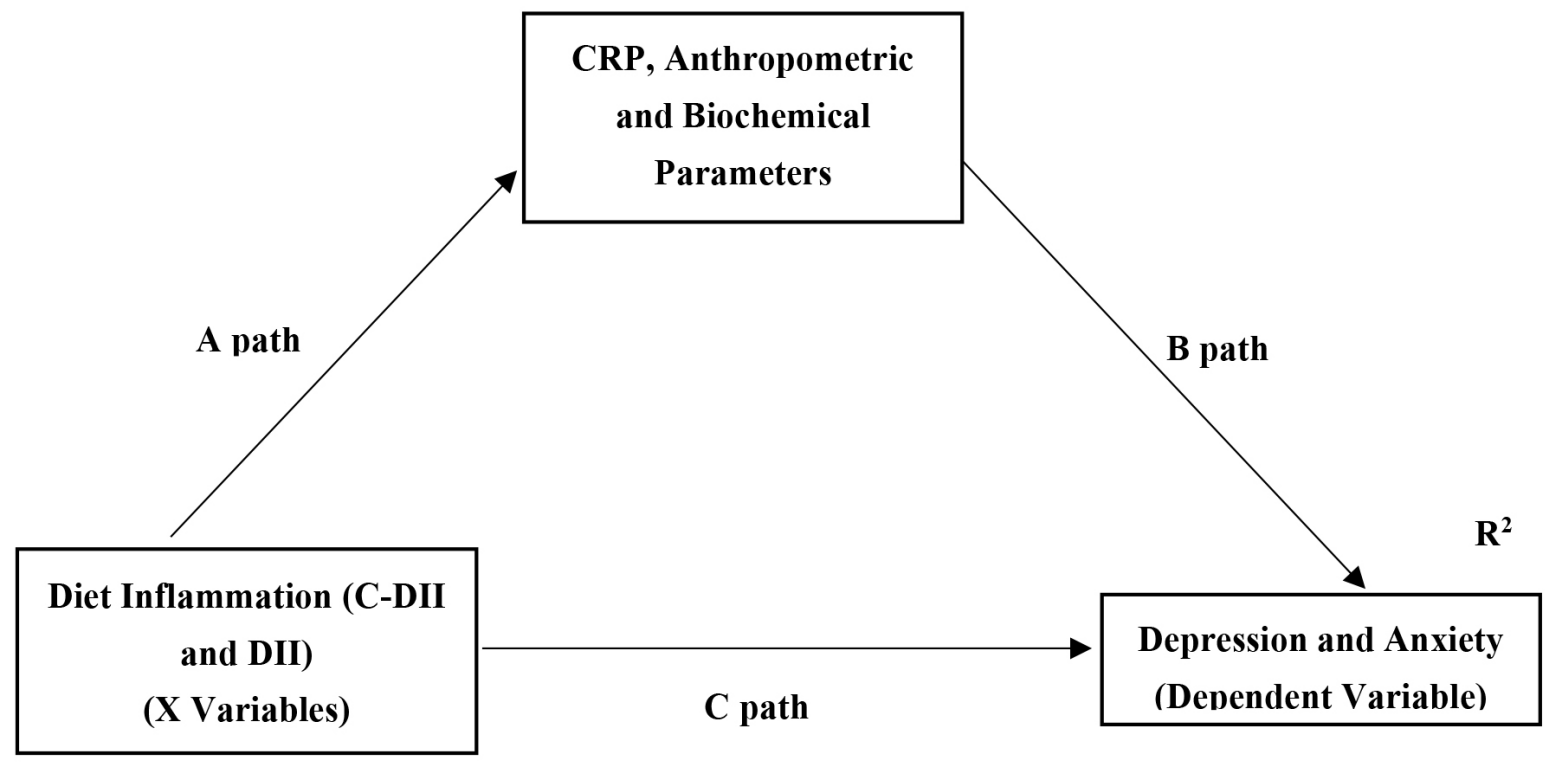
**Hypothesized model in which anthropometric and biochemical parameters and inflammation marker as a mediating variable diet inflammation to depression and anxiety**. C-DII and DII express the independent variables and depression and anxiety scores express the dependent variables in the model. CRP, anthropometric and biochemical parameters represent the mediator effect of the independent variable on the dependent variable. Paths (A), (B) and (C) indicate the linear regression path coefficients of each on the other variable. The total effect on the dependent variable is obtained by summing the indirect effect (A×B) and the path coefficient (C) of the independent variable on the dependent variable with the direct effect. R^2^ represents the coefficient of explanation of both independent and mediator variables on the dependent variable. C-DII, Children-dietary inflammatory index; CRP, C-reactive protein; DII, dietary inflammatory index. Data are standardised regression weights (β) for paths.

Structural equation models (SEMs) were applied to assess the proposed theoretical models (Fig. 1). In this model, the dependent variables were depression (CDI) and anxiety (SASC-R), and the independent variables were dietary inflammation status (C-DII and DII). In the first step, conceptual models were developed on the basis of information obtained from the correlation matrix table. Anthropometric measurements, biochemical parameters, and inflammation markers associated with the dependent and independent variables were included in the SEM analysis. In this context, we examined the mediating role of some variables associated with cardiometabolic risk factors in the relationship between depression and anxiety and dietary inflammation. In the path analysis, a standardized path coefficient was used to compare the effects of the independent variables on the dependent variables. The total, direct, and indirect effects, 95% confidence intervals, and explanation coefficients are presented in the SEM analysis. Bootstrapping (5000 replications) was used to generate normal-based bootstrapped confidence intervals around the indirect effect. If the total effect is statistically significant and the confidence interval of the indirect effect does not include zero, then there is a mediation effect. Partial mediation occurs if the direct effect is statistically significant; otherwise, full or complete mediation. To verify the fit of the model, some measurements were analyzed: chi-square fit statistics/degree of freedom (CMIN/DF) value <2, indicating a reasonable fit; root mean square error of approximation (RMSEA) and standardized root mean square residual (SRMR), with values <0.08 and <0.10, respectively indicating that the theoretical model fits the data; Tucker–Lewis index (TLI) >0.95 and comparative fit index (CFI) and goodness-of-fit statistic (GFI), where values >0.90 indicate a good fit of the model. *p*-values less than 0.05 were considered statistically significant [26].

## 3. Results

In this cross-sectional study, 52% of the participants were girls, and the mean age of the participants was 11.5 ± 0.08 years, with similar mean age and gender distributions across the C-DII tertiles. The BMI-z score was statistically higher in the pro-inflammatory group than in the anti-inflammatory group (0.85 ± 0.15 vs. 1.13 ± 0.15, respectively; *p* > 0.05). It was determined that waist circumference, Waist-to-height ratio (WHtR), and body weight were higher in the increasing tertile 3 (most pro-inflammatory) group than in tertile 1 (most anti-inflammatory). Increased dietary inflammation burden was associated with higher depression and anxiety scores (Tertile1 vs. tertile 3; 23.5 ± 0.27 and 25.5 ± 0.25 for depression; 41.0 ± 1.30 and 46.4 ± 1.11 for anxiety, *p* < 0.05) (Table 1).

**Table 1.** **Characteristics of study participants across tertiles of the C-DII score in adolescents**.

Variables	Total (n = 304)		Tertile 1 (n = 101)		Tertile 2 (n = 102)		Tertile 3 (n = 101)		*p* value
	mean	SE	mean	SE	mean	SE	mean	SE	
C-DII score	7.38	0.10	5.23	0.10	7.54	0.04	9.36	0.07	-
DII score	3.21	0.06	1.95	0.07	3.30	0.03	4.38	0.06	-
Gender (girls)									
n	158		55		55		57		0.268^a^
%	52.0		45.5		54.9		56.4		
Age (y)	11.5	0.08	11.6	0.14	11.4	0.14	11.6	0.15	0.520^b^
Body weight (kg)	48.9	0.84	46.8	1.38	47.6	1.38	51.2	1.52	0.037^b⁣*^
BMI (kg/m^2^)	21.4	0.37	20.8	0.45	21.8	0.51	22.1	0.67	0.018^b⁣*^
BMI-z score	0.97	0.08	0.85	0.15	0.89	0.15	1.13	0.15	0.026^b⁣*^
WC (cm)	73.8	0.76	72.5	1.26	74.5	1.30	76.5	1.38	0.044^b⁣*^
Waist height ratio	0.48	0.00	0.47	0.00	0.46	0.00	0.51	0.00	0.021^b⁣*^
BFG (mg/dL)	89.1	0.61	87.3	1.12	88.2	0.99	90.7	1.03	0.587^b^
Triglyceride (mg/dL)	122.6	11.5	122.5	16.4	131.0	30.1	117.5	9.41	0.893^b^
LDL-C (mg/dL)	97.3	3.16	96.9	4.65	89.1	5.78	102.8	5.40	0.210^b^
HDL-C (mg/dL)	44.9	1.11	47.1	1.60	45.3	1.79	43.1	2.42	0.032^b⁣*^
CRP (mg/L)	0.05	0.01	0.01	0.00	0.04	0.01	0.10	0.02	0.014^b⁣*^
Albumin (g/dL)	4.85	0.02	4.82	0.07	4.88	0.03	4.86	0.02	0.661^b^
CDI	24.2	2.5	23.5	0.27	24.0	0.29	25.5	0.25	0.020^b⁣*^
SASC-R	45.1	0.72	41.0	1.30	45.8	1.25	46.4	1.11	0.007^b^

BFG, Blood fasting glucose; BMI, Body mass index; CRP, C-reactive protein; CDI, Child Depression Inventory; C-DII, Children’s dietary inflammatory index; DII, Dietary inflammatory index; HDL-C, High-density lipoprotein cholesterol; LDL-C, Low-density lipoprotein cholesterol; SASC-R, Social anxiety scale for children-revised form; WC, waist circumference; SE, standard error. ^a^ Chi-square test. ^b^ One-way analysis of variance (ANOVA) test. ^*^p < 0.05.

In all participants, high C-DII scores were independently associated with high CDI score [β (95% CI) = 0.224 (0.08–0.25), *p* < 0.05]. In addition, this relationship was maintained in both gender groups. Similar results were found for anxiety, where β was 0.923 (95% CI 0.48–1.67, *p* < 0.05) for all participants and 1.151 (95% CI 0.12–0.52, *p* < 0.01) for boys but not among girls (Table 2).

**Table 2.** **Estimated beta and standard errors for the effects of children-dietary or dietary inflammatory index quartiles and total depression and anxiety score**.

Dependent Variables	Participants	C-DII				DII			
		β	SE	95% CI	*p* value	β	SE	95% CI	*p* value
CDI	All (n = 304)	0.224	0.05	0.08–0.25	0.032^*^	0.250	0.07	0.16–0.29	0.020^*^
	Boys (n = 146)	0.278	0.07	0.09–0.28	0.015^*^	0.269	0.10	0.04–0.34	0.012^*^
	Girls (n = 158)	0.197	0.04	0.09–0.26	0.044^*^	0.223	0.09	0.12–0.31	0.037^*^
SASC-R	All (n = 304)	0.923	0.38	0.04–1.67	0.010^*^	1.141	0.52	0.08–2.36	0.008^**^
	Boys (n = 146)	1.151	0.52	0.12–2.18	0.009^**^	1.511	0.75	0.18–3.20	0.003^**^
	Girls (n = 158)	0.268	0.56	–0.85–1.38	0.472	0.684	0.90	–1.09–2.46	0.449

Beta coefcient, standard error, 95% confidence interval and p value calculated from linear regression analysis. ^*^p < 0.05, ^**^p < 0.01.

According to the correlation matrix results, there was a moderate negative association between C-DII and CRP levels (r = 0.318), but a weak association for HDL-c, BMI-z score, WC, and WHtR (r = –0.239, 0.242, 0.253 and 0.237, respectively; *p* < 0.05). C-DII and DII were positively associated with depression and anxiety. Moreover, depression scores were weakly positively associated with CRP levels, BMI-z score, WC and WHtR. Anxiety was only correlated with CRP and HDL-c levels (Table 3). If the independent (C-DII and DII) and dependent variables (depression and anxiety) were found to be statistically significant with biochemical and anthropometric measurement parameters, they were included in the SEM analysis as mediator variables. In this context, we investigated the mediating role of BMI-z score, WC, and WHtR for the association between dietary inflammation and depression, and CRP and HDL-c levels for anxiety.

**Table 3.** **Correlation matrix output between dietary inflammation, depression, anxiety, anthropometric measurements and biochemical parameters**.

Variables	1.	2.	3.	4.	5.	6.	7.	8.	9.	10.	11.	12.	13.
1. C-DII	-												
2. DII	0.927^*^	-											
3. CDI	0.207^*^	0.215^*^	-										
4. SASC-R	0.257^*^	0.279^*^	0.249^*^	-									
5. CRP	0.318^*^	0.309^*^	0.276^*^	0.213^*^	-								
6. BFG	0.032	0.060	0.015	–0.030	0.077	-							
7. Total-c	0.057	0.060	0.110	0.061	–0.095	0.043	-						
8. Triglyceride (mg/dL)	0.006	0.017	0.117	0.012	–0.008	–0.263^*^	0.574^*^	-					
9. LDL-C (mg/dL)	0.056	0.029	0.019	0.048	–0.017	0.041	0.891^*^	0.343^*^	-				
10. HDL-C (mg/dL)	–0.239^*^	–0.218^*^	0.053	–0.254^*^	0.060	–0.257^*^	0.224^*^	–0.421^*^	–0.009	-			
11. BMI-z score	0.242^*^	0.243^*^	0.295^*^	0.082	–0.017	0.037	0.267^*^	0.016	0.401^*^	–0.259	-		
12. WC (cm)	0.253^*^	0.269^*^	0.278^*^	0.028	0.024	–0.093	0.230^*^	0.043	0.363^*^	–0.352^*^	0.834^*^	-	
13. Waist height ratio	0.237^*^	0.231^*^	0.261^*^	0.061	0.010	–0.101	0.358^*^	–0.007	0.419^*^	–0.230	0.878^*^	0.916^*^	-

BFG, Blood fasting glucose; BMI, Body mass index; CRP, C-reactive protein; CDI, Child Depression Inventory; C-DII, Children’s dietary inflammatory index; DII, Dietary inflammatory index; HDL-C, High-density lipoprotein cholesterol; LDL-C, Low-density lipoprotein cholesterol; SASC-R, Social anxiety scale for children-revised form; WC, waist circumference. Pearson’s correlation test. ^*^p < 0.05.

Table 4 shows the mediating effect of anthopometric measures, CRP, and HDL-c levels on the association between dietary inflammation and depression and anxiety. The total effect of C-DII on depression and anxiety was statistically significant (β [95% CI] = 0.224 [0.08–0.25]; 0.727 [0.10–1.33], *p* < 0.05, respectively). With the exception of HDL-c for anxiety and WHtR for depression, other variables were found to have mediating effects. CRP and WC partially mediated the relationship between C-DII and depression, and the coefficient of explanation of depression by independent variables was 18.6% for CRP and 15.6% for WC. The association between C-DII and depression was explained by CRP (23.2%) and WC (20.9%) as mediator variables (indirect effect β coefficient/total effect β coefficient). The CRP level was found to partially mediate the association between C-DII and anxiety, but not for HDL-c levels [indirect effect (95% CI)] = 0.104 [0.06–0.15] for CRP). CRP level and C-DII score explained 20.4% (R^2^) of the change in anxiety. It was found that 14.3% of this coefficient of explanation came from indirect effects. In other words, approximately 15% of the association between C-DII and anxiety was explained by CRP in our model. In the SEM analysis, the indirect, direct, and total effects of DII on depression and anxiety were similar to those of C-DII. The goodness-of-fit indices for each SEM analysis are presented in detail (**Supplementary Table 1**). The goodness-of-fit indices for the final structural models indicated a good fit.

**Table 4.** **Direct, indirect, and total effects of diet inflammation on depression and anxiety mediated by some anthropometric incidences and biochemical findings**.

Dependent variable	Mediators	Associations	Independent variable							
			C-DII				DII			
			β	SE	95% CI	R^2^	β	SE	95% CI	R^2^
CDI	CRP	Direct	0.172	0.04	0.09–0.24	0.186	0.179	0.03	0.13–0.22	0.151
		Indirect	0.052	0.01	0.01–0.04		0.071	0.01	0.02–0.10	
	BMI-z score	Direct	0.196	0.05	0.15–0.24	0.127	0.213	0.04	0.17–0.25	0.132
		Indirect	0.028	0.00	0.01–0.04		0.037	0.00	0.01–0.07	
	WC	Direct	0.178	0.03	0.15–0.20	0.156	0.158	0.03	0.11–0.21	0.148
		Indirect	0.047	0.01	0.02–0.07		0.092	0.01	0.05–0.14	
	WHtR	Direct	0.215	0.06	0.16–0.22	0.116	0.233	0.06	0.19–0.27	0.105
		Indirect	0.009	0.00	–0.02–0.03		0.017	0.00	–0.01–0.02	
SASC-R	CRP	Direct	0.623	0.19	0.24–0.11	0.204	0.950	0.30	0.61–1.48	0.235
		Indirect	0.104	0.01	0.06–0.15		0.190	0.08	0.12–0.27	
	HDL-c	Direct	0.689	0.16	0.27–1.15	0.177	1.092	0.24	0.82–1.53	0.184
		Indirect	0.038	0.00	–0.02–0.08		0.048	0.01	–0.03–0.11	

BFG, Blood fasting glucose; BMI, Body mass index; CRP, C-reactive protein; CDI, Child Depression Inventory; C-DII, Children’s dietary inflammatory index; DII, Dietary inflammatory index; SASC-R, Social anxiety scale for children-revised form; WC, waist circumference. Total-C, Total cholesterol.

Mediation analyses were conducted through linear regression using the IBM SPSS AMOS version 26. A bootstrap method using iterations of computed samples (5000) was used to determine the significance of the indirect effects. All paths were given standardized regression path coefficients. Except for the mediating effect of HDL in the effect of C-DII and DII on anxiety, all pathways were statistically significant.

## 4. Discussion

In this cross-sectional study, we found significant associations between dietary inflammation and anxiety and depression scores in all adolescent participants. Except for dietary inflammation with anxiety in girls, these relationships remained statistically significant in all subgroups by sex. It was determined that CRP partially mediated the relationship between dietary inflammation and depression and anxiety. Our findings showed that the BMI-z score and WC mediated the relationship between dietary inflammation and depression levels; thus, both inflammatory mediators (CRP levels) and anthropometric measurements (WC and BMI-z score) could explain part of the association between depression and diet inflammation.

The depression score was lower in the anti-inflammatory group than in the other groups in this study, and this relationship remained in the linear regression analysis. Currently, meta-analysis review has confirmed this relationship [27]. Shivappa* et al*. [28] included Iranian adolescents in their study and did not find a linear regression relationship between depression symptoms score and DII after full adjustment (β (95% CI) = 1.67 (0.40–3.31), *p* < 0.05). In another study design, adolescents aged 14–17 years were included, and western dietary patterns were associated with increased mental health problems, resulting in higher inflammation and formation of adipose tissue [29]. Conversely, reverse causality may be observed in the relationship between depression and diet. Therefore, increased depressive symptoms may increase the inflammatory burden of the diet by increasing the tendency of adolescents to eat more unhealthy diets. The results of some studies also support this view [30].

Our findings support the hypothesis that C-DII and DII are associated with the occurrence of anxiety. Studies in Brazil [31] and Iran [32] were shown to be associated with an increased risk of anxiety in the last quartiles (more reflection of a pro-inflammatory diet) compared with the reference group (Odds Ratio (OR) (95% CI) = 1.37 (1.03–1.83; 1.60 (1.15–2.24), *p* < 0.01, respectively)). However, there was no association between DII and anxiety in either men or women after stratification by sex. In contrast to these research findings, our results on the relationship between DII and anxiety remained significant only among boys. We believe that diet may play a minor role in the development of mental health problems such as anxiety in women, as hormonal fluctuations may play a more effective role. However, the presence of estrogen hormone in women can have a protective effect on humoral immunity, and accordingly may also have a protective effect on chronic diseases and mental disorders [33]. Therefore, the intake of an anti- or pro-inflammatory dietary pattern could have no effect on anxiety in girls.

Pro-inflammatory diets can increase the permeability of the intestinal barrier, resulting in a leaky gut and thus causing bacterial translocation that can lead to depression and anxiety [34]. Recently, it has been suggested that an increase in the level of inflammation may play a role in the development of depression and anxiety according to the leaky gut theory. Plasma immunoglobulin levels (especially IgA and IgM) increase in response to lipopolysaccharides produced by gram-negative bacteria in the intestinal flora [35]. In addition, an increase in this immune response can increase the cascade formation of molecules such as nuclear factor kappa beta (NF-κB), which causes the activation of proinflammatory cytokines (TNF-α, IL-2 and COX-2). The formation of oxidative stress factors accelerates these metabolic pathways. All of these mechanisms cause a vicious circle, accelerating and exacerbating the occurrence of depression and anxiety [36]. Moreover, cytokine formation can stimulate the HPA axis, leading to increased release of stress hormones. Various dietary patterns can affect anxiety through changes in neurotransmitter health, oxidative stress, and HPA axis [37]. It has been reported that inflammation could play a role in the relationship between diet quality and mental disorders. In the Nursing Health Study and the Health Professionals Follow-up Study, a positive relationship was observed between western diet consumption and CRP and IL-6 levels in all gender groups [38]. In our study, SEM analysis showed that the indirect effect of diet inflammation status on depression and anxiety on CRP levels was consistent with the mentioned mechanisms. Azarmanesh* et al*. [18] showed in a cross-sectional study that the relationship between DII and depression was partially mediated through CRP, with an explanation coefficient of 3.6%.

Metabolic risk components such as lipid profile, blood sugar, and abdominal obesity affect the formation of mental disorders in various ways, as well as inflammation [39]. If the factors that cause metabolic risk factors are prevented in adolescence or childhood, both chronic diseases and mental disorders can be prevented in adulthood and older age. In this case, it is crucial to explain the effect of metabolic risk factors on the relationship between the inflammatory load of the diet and anxiety and depression. In structural equation modeling, only the WC and BMI-z score played a partial mediation role in the relationship between the inflammatory state of diet and depression. In a study on Australian adolescents, the western diet pattern (i.e., high amounts of red meat, processed and refined foods, sweets, etc.) was found to be associated with higher BMI, waist circumference, depressive and anxiety symptoms, and CRP and leptin levels [29]. In the formation of metabolic risk factors, the first expected development is central and abdominal obesity. Subsequently, it can lead to the development of metabolic disorders by causing an increase in inflammatory and adipokine levels, leading to the disruption of carbohydrate and lipid metabolism in the body [40]. Therefore, biochemical parameters may not have had an indirect effect on the relationship between mental health and dietary inflammation. In this case, it is necessary to follow the participants for a long time to reveal the effect of the relationship.

To the best of our knowledge, this study is the first to examine the influence of inflammation and metabolic risk factors between diet inflammatory status and mental status in adolescents. Both C-DII and DII were used to calculate the inflammatory load of the diet, and their relationships with all parameters were tested simultaneously. The CDI and SASC-R scales used to evaluate depressive and anxiety symptoms have validity and reliability.

There are several limitations to this study. Some components were missing and were used in the calculation of DII and C-DII. However, the number of components used in the calculation of the index is similar to that in previous studies [41, 42]. No clinical diagnosis of depression or anxiety was made. Therefore, if depression and anxiety are clinically diagnosed in the future, it will help to minimize possible misclassification of results. This was a cross-sectional study; therefore, we were unable to infer causality from the associations of DII with CRP, BMI-z score, waist circumference, depression, or anxiety. An unhealthy diet and inflammation can exacerbate depression, but the tendency of an unhealthy diet has increased because of depression, and thus inflammation may have developed as a result. In particular, well-designed longitudinal studies are required to avoid this reverse causality. It may not be sufficient to investigate the effect of only one inflammation marker on the relationship between depression and nutrition; instead it should also be investigated whether more than one inflammation marker has a mediating effect. Lastly, the number of study samples was quite small; therefore, we could not adjust for confounding factors that may play a role in the research hypotheses.

## 5. Conclusions

In conclusion, our results indicate that a diet with a high pro-inflammatory potential is associated with a higher risk of depression and anxiety in adolescents, and inflammation markers as assessed by CRP levels are one of the main mediators that may occur. However, obesity as assessed by BMI-z score and WC is one of the main mediators that may occur in the relationship between diet inflammation and depression. These results help to improve our understanding of the mechanisms underlying diet-related inflammation and depression in adolescents. Therefore, adolescents who may be at risk of depression or anxiety should be encouraged to consume more anti-inflammatory foods and fewer pro-inflammatory foods. Future studies, especially prospective studies, are urgently needed to validate the association between diet and depression and anxiety from the perspective of inflammation and cardiometabolic risk factors in adolescents, which might provide useful dietary interventions for the prevention and treatment of mental disorders.

## Availability of Data and Materials

The datasets generated and analyzed during the current study are not publicly available due to privacy and ethical restrictions involving participant data but are available from the corresponding author on reasonable request. Requests for data access will be considered by the authors in line with institutional and ethical guidelines, ensuring the protection of participants’ confidentiality.

## Supplementary Material

Supplementary material associated with this article can be found, in the online version, at https://doi.org/10.31083/AP38791.
