# Supplementary material for: Association of Children’s Dietary Inflammatory Index with Depression and Anxiety Symptoms in Adolescents: Mediating Role of Inflammation and Cardiometabolic Risk Factors
Source: Alpha Psychiatry. 2025 Feb 28;26(1):38791. doi: 10.31083/AP38791 (PMC11915713; doi:10.31083/AP38791)
Supplement: Supplementary file 1 [file 2757-8038-26-1-38791-s1.docx]

**SUPPLEMENTARY FILE**

**Supplementary Table 1.** Goodness of fit indices for structual equation models

| Relationship | Mediators | χ2/df  (<2.00) | GFI  (>0.90) | CFI  (>0.90) | TLI  (>0.95) | RMSEA  (<0.08) | SRMR  (<0.10) |
| --- | --- | --- | --- | --- | --- | --- | --- |
| C-DII Depression | CRP | 0.476 | 1.000 | 0.994 | 1.000 | 0.015 | 0.032 |
|  | BMI-z score | 0.783 | 0.929 | 0.981 | 1.000 | 0.043 | 0.035 |
|  | WC | 0.154 | 1.000 | 1.000 | 0.974 | 0.056 | 0.019 |
|  | WHtR | 0.562 | 0.972 | 0.987 | 0.951 | 0.039 | 0.038 |
| DII Depression | CRP | 0.350 | 0.997 | 1.000 | 0.984 | 0.032 | 0.024 |
|  | BMI-z score | 0.550 | 0.942 | 0.956 | 1.000 | 0.062 | 0.072 |
|  | WC | 1.413 | 1.000 | 1.000 | 0.975 | 0.045 | 0.075 |
|  | WHtR | 1.515 | 0.943 | 1.000 | 0.972 | 0.061 | 0.083 |
| C-DII Anxiety | CRP | 1.383 | 0.917 | 0.965 | 1.000 | 0.036 | 0.062 |
|  | HDL-c | 1.410 | 0.937 | 0.968 | 1,000 | 0.037 | 0.041 |
| DII Anxiety | CRP | 1.388 | 1.000 | 1.000 | 0.980 | 0.029 | 0.060 |
|  | HDL-c | 0.669 | 0.951 | 0.960 | 1.000 | 0.038 | 0.071 |

χ2/df= Chi-Square value/Degrees of freedom; CFI= Comparative fit index; GFI= goodness-of-fit index; RMSEA= Root mean square error of approximation; SRMR= Standardized root mean square residual; TLI= Tucker–Lewis index.

It shows the goodness-of-fit values for the SEM analyses in Table 4. All goodness-of-fit values for each model were at acceptable levels. Therefore, modification index for models was not required.
